# Adult Nephroblastoma with Predominant Epithelial Component: A Differential Diagnostic Candidate of Papillary Renal Cell Carcinoma and Metanephric Adenoma—Report of Three Cases

**DOI:** 10.1155/2013/675875

**Published:** 2013-09-03

**Authors:** Shiho Watanabe, Hiroshi Naganuma, Michio Shimizu, Satoshi Ota, Shin-ichi Murata, Naoki Nihei, Jun Matsushima, Shuji Mikami, Naoto Kuroda, Yoji Nagashima, Yukio Nakatani

**Affiliations:** ^1^Departments of Diagnostic Pathology, Chiba University Graduate School of Medicine, Chiba 260-8670, Japan; ^2^Divisions of Surgical Pathology, Sendai City Hospital, Sendai 984-8501, Japan; ^3^Department of Pathology, Saitama Medical University International Medical Center, Saitama 350-1298, Japan; ^4^Department of Human Pathology, Wakayama Prefectural Medical College, Wakayama 641-8509, Japan; ^5^Department of Urology, Chiba University Graduate School of Medicine, Chiba 260-8670, Japan; ^6^Division of Diagnostic Pathology, Keio University Hospital, Tokyo 160-8582, Japan; ^7^Division of Diagnostic Pathology, Kochi Red Cross Hospital, Kochi 780-8562, Japan; ^8^Department of Molecular Pathology, Yokohama City University Graduate School of Medicine, Yokohama 236-0004, Japan

## Abstract

Although nephroblastoma is the commonest renal tumor of childhood, it is rare in adults. In cases of predominantly epithelial type occurring in adulthood, it might be difficult to distinguish it from papillary renal cell carcinoma and metanephric adenoma. Here, we report three cases of adult epithelial nephroblastoma in 24-, 76-, and 21-year-old females. Histologically, the tumors were composed of papillotubular architectures of small and uniform tumor cells with high nucleocytoplasmic ratio without blastemal element. Immunohistochemically, the tumor cells were positive for WT-1 and CD57 but negative for AMACR, which was helpful to exclude the possibility of papillary renal cell carcinoma. Metanephric adenoma is a benign tumor, which can be distinguished by the observation of the cellular atypism and growth pattern. However, nephroblastoma with predominant epithelial element mimics the malignant counterpart of metanephric adenoma, that is, “metanephric adenocarcinoma.”

## 1. Introduction

Nephroblastoma (Wilms tumor) is the most frequent renal tumor in childhood and accounts for 6%-7% of pediatric tumors. Nephroblastoma is most frequent in the first decade, and generally the initial presentation is abdominal distention. Histologically, nephroblastoma is composed of blastemal, epithelial, and stromal elements in various proportions. The blastemal element is densely packed and oval to spindle undifferentiated cells. The epithelial element forms abortive tubular and glomerulus-like structures. The stromal element is more or less differentiated nonepithelial cells, occasionally showing differentiation to the striated muscle, bone, and cartilage [1]. In cases that nephroblastoma is composed of a dominant epithelial element, differential diagnosis from papillary RCC, metanephric adenoma, and other renal tumors is challenging, especially in adult cases. 

Here, we report three cases of adult nephroblastoma with overwhelming epithelial element. We made comparison of immunohistochemical characteristics with metanephric adenomas, nephroblastoma, and conventional papillary RCCs and revealed that these are nephroblastoma with extremely epithelial element. In cases of scarce blastemal element, they are considered as the malignant counterpart of metanephric adenoma, “metanephric adenocarcinoma.” Such a tumor should be distinguished from papillary RCC and metanephric adenoma.

## 2. Case Reports

**Case 1**

The patient was a 24-year-old Japanese female, who had developed gross hematuria. Her family or past history was not contributory. The general status or laboratory data was not remarkable. Radiological examination showed a tumorous lesion in the upper pole of the right kidney, along with enlarged renal hilar nodes. Other than the renal tumor, there was no possible lesion as the primary site. Under a clinical diagnosis of RCC, right radical nephrectomy was performed. However, the tumor recurred in the retroperitoneum and peritoneal cavity, and the patient died, 18 months after the surgery. Autopsy was not performed.

**Figure 1:**
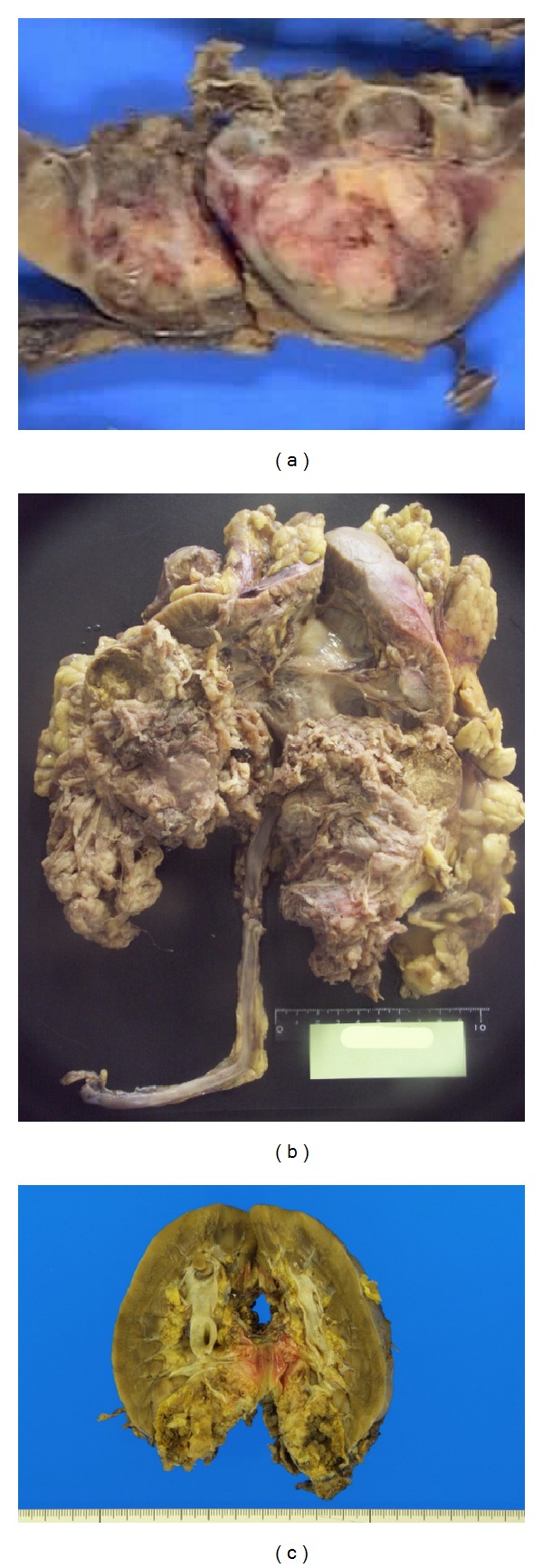
Gross finding of the tumor. (a) The tumor occupied the upper pole of the kidney. The cut surface was mottled with whitish color and hemorrhage (Case 1). (b) The resected kidney contained a brownish and fragile tumor in the lower half (Case 2). (c) The tumor was milky white in color and occupied the lower pole of the kidney (Case 3).

The surgically resected kidney measured 12 × 8 × 2 cm in size and contained a tumor measuring 3 cm in diameter, in the upper pole. The tumor was well demarcated from the renal parenchyma without capsule formation. Invasion to the perirenal fat or renal sinus was absent. The cut surface of the tumor was mottled with grayish white and dark red colors (Figure 1(a)). Hemorrhage and necrosis were also observed. The vascular or ureteric margins were not involved.

**Case 2**

The patient is a 76-year-old Japanese female, who had developed gross hematuria. Radiological examination revealed a huge tumor in the left kidney with para-aortic nodal enlargement. Under the clinical diagnosis of RCC with nodal metastasis, radical nephrectomy and lymphadectomy were performed. The patient is doing well, 4 months after the operation.

The resected kidney contained a fragile grayish brown tumor filling the lower half of the pelvic cavity, measuring approximately 10 cm in maximal diameter. The tumor was protruded from the renal parenchyma. The boundary was poorly demarcated. The upper half of the pelvis was dilated due to urinary outflow block by the tumor (Figure 1(b)).

**Case 3**

The patient is a 21-year-old female, who had developed right-sided abdominal pain and referred to the hospital. Radiological examination revealed a tumorous lesion in the right kidney. Once, the tumor was diagnosed as metanephric adenoma, with needle biopsy under CT guiding, and the patient was followed up. However, the tumor had presented enlargement, and the right kidney was totally resected. The patient is doing well for 8 months after the surgery.

The resected kidney contained an infiltrative tumor, measuring 5 cm in diameter, in the lower pole. The cut surface of the tumor was milky white in color and fragile in consistence (Figure 1(c)).

### 2.1. Histopathological Findings

**Figure 2:**
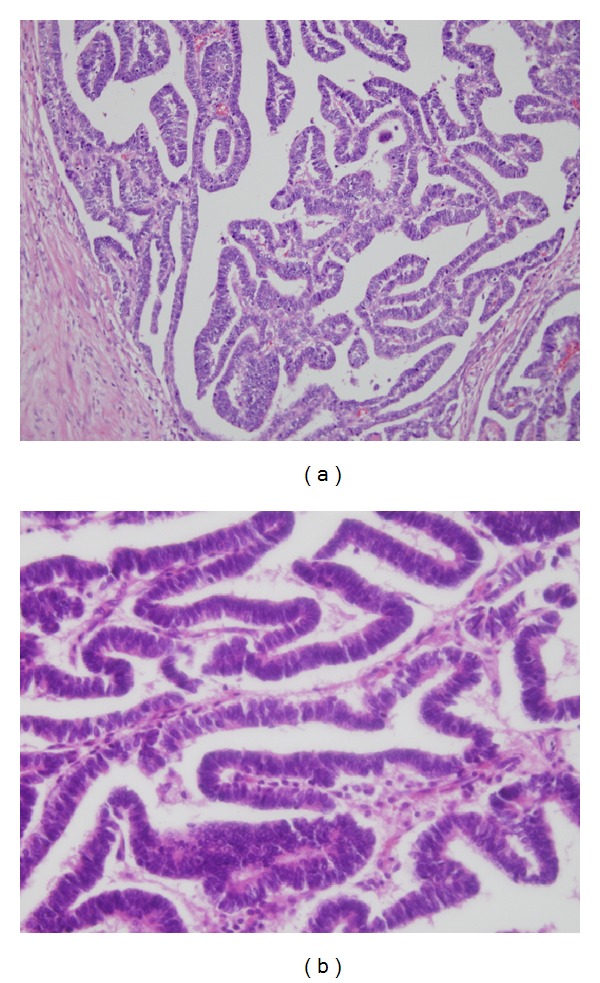
Microscopic findings of the tumor. (a) The tumors were composed of papillotubular architectures with an invasive growth. The stroma showed hyalinization. (b) The tumor cells were high columnar epithelia. The nuclei were oval in shape with fine chromatin and inconspicuous nucleoli. The cytoplasm was scanty.

All the tumors showed an invasive growth into the renal parenchyma, without pseudocapsule. All the tumors were composed of papillotubular architectures with a hyalinized stroma (Figure 2(a)). The tumor cells were uniform, small in size, and columnar in shape. The nucleocytoplasmic ratio was high. Their nuclei were spherical in shape and contained fine chromatin. The cytoplasm was scanty (Figure 2(b)). Psammoma bodies were absent. All the tumors are of pure epithelial in nature, lacking blastemal and stromal elements.

**Figure 3:**
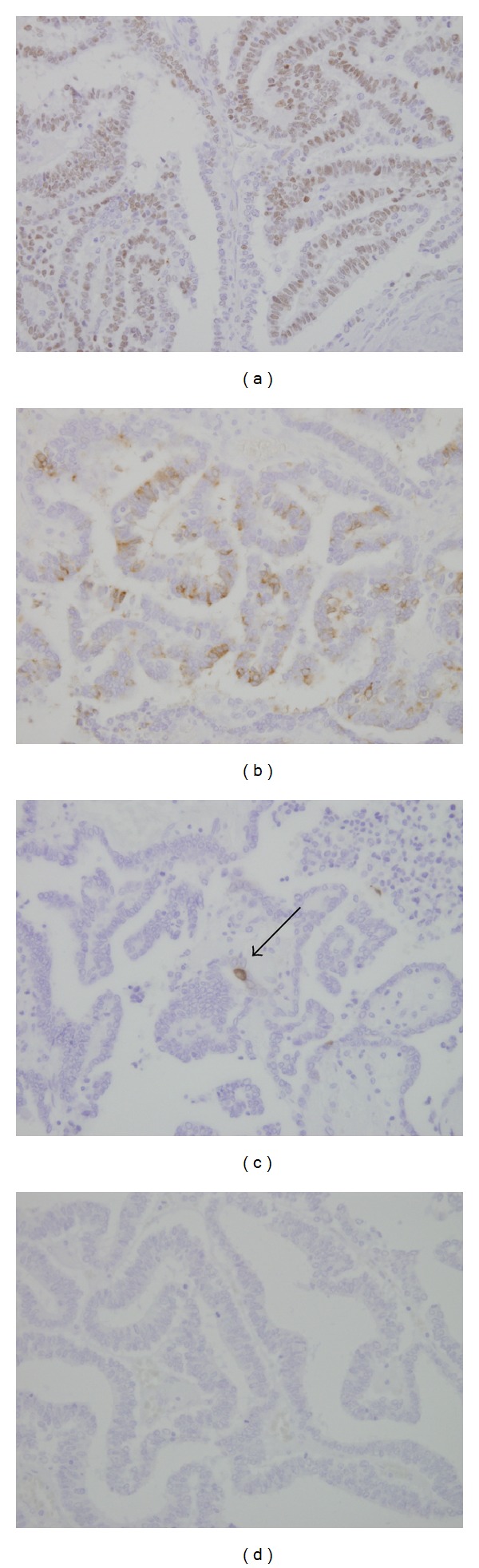
Immunohistochemical findings of the tumor. (a) The tumor cells were positive for WT-1. (b) The tumor cells were positive for CD57. (c) The tumor cells were occasionally positive for CK7 (arrow). (d) AMACR failed to be detected in the tumor cells.

Immunohistochemically, the present tumor cells were partly positive for epithelial membrane antigen (EMA) but not vimentin. Pan-cytokeratin (detected with clone AE1/AE3) was diffusely positive. The tumor cells were positive for WT-1 (Figure 3(a)) and focally positive for CD57 (Figure 3(b)) but negative for AFP. A few tumor cells were positive for cytokeratin 7 (CK7) (Figure 3(c)) and CK20. CD10, alpha-methylacyl-CoA racemase (AMACR) (Figure 3(d)), or RCC-MA was not detected. Neuroendocrine differentiation markers, chromogranin A, synaptophysin, and CD56 were negative. Thyroid transcription factor-1 (TTF-1) was also negative.

## 3. Discussion

Although its majority is clear cell RCC, renal neoplasms contain various subtypes. Adopting recent knowledge on genetic features, its classification has been revised, involving several novel histological types, that is, mucinous tubular and spindle cell carcinoma and Xp11.2/*TFE3 *translocations-associated RCC [2].

Here, we present three cases of aggressive renal tumors composed of papillotubular architectures. The tumor cells were small in size and uniform in shape. Because of high nucleocytoplasmic ratio, we suspected the embryonal nature of the tumor cells, although they lacks the nephroblastic element. Consequently, we performed immunohistochemical staining for WT-1 and CD57, both of which are the markers of developing nephron as well as metanephric tumors. As expected, both markers showed positive reactions. Based on these results, we considered the lesion as an epithelial malignant tumor with embryonal characteristics.

As candidates of differential diagnoses, conventional papillary RCC, mucinous tubular spindle cell carcinoma (MTSCC), Xp11.2/*TFE3* translocations-associated RCC, nephroblastoma (especially epithelial type), neuroendocrine carcinoma, and metastatic thyroid papillary carcinoma should be evaluated.

Papillary RCC is the second most frequent type of RCCs. The tumor is dominantly composed of papillotubular architectures. Based on the cellular atypism, the tumor is subclassified into type 1 (small-sized tumor cells with low nuclear grade) and type 2 (relatively large-sized tumor cells with higher nuclear grade) [3]. The present cases were relatively similar to type 1. However, papillary RCC are negative for WT-1 and CD57, and the present cases were negative for AMACR [4]. Previously, we found that immunoreactivity for AMACR is useful to distinguish papillary RCC from collecting duct carcinoma [5]. Additionally, CK7 is diffusely positive in most of papillary RCCs [6], whereas the present case showed positivity in limited cell population. Together with these results, the present case has characteristics different from the conventional papillary RCC. Because trisomies of chromosomes 7 and 17 are frequent in papillary RCC, absence of these abnormalities might be helpful for differential diagnosis, although we did not perform cytogenetic study.

MTSCC is a newly recognized subtype and is composed of slender and elongated tubules and fascicle of the spindle cells, with a mucinous stroma [7]. The tumor cells are small in size and uniform. Their nuclei are small round with fine chromatin. MTSCC is frequent in middle-aged female. Although most cases of the tumor are indolent, there are several reports of cases with high nuclear grade and aggressive course. MTSCC shows positivity of AMACR, which suggests some relationships with papillary RCC [7]. Considering the patient's gender and uniform and small-sized tumor cells, MTSCC might be a candidate of differential diagnosis. However, the present tumor was entirely composed of papillary architectures, and the spindle element and mucinous stroma were absent. Immunohistochemical staining showed no reactivity with AMACR. Therefore, the possibility of MTSCC can be excluded.

Xp11.2/*TFE3* translocations-associated RCC is a newly recognized subtype of RCC, which bears chromosomal translocation involving Xp11.2 [8]. The tumor predominantly occurs in children and young adults. Histologically, the tumor is composed of papillary architecture and solid cell nests of high grade tumor cells. Psammoma bodies are frequently seen. Characteristically, the translocation RCC shows aberrant nuclear immunoreactivity for *TFE3*, the product of *TFE3* gene located on Xp11.2 [9]. Although the present case occurs in a young adult, the histological appearance is not typical as Xp11.2/*TFE3* translocations RCC. Further, TFEB translocations-associated RCC has been recognized as a newly subtype. This subtype is characterized by nested morphology and immunohistochemical positivity of TFEB [10], which were not observed in the present cases.

Based on small and uniform cells with a hyalinized stroma, the possibility of neuroendocrine carcinoma should be evaluated. Immunohistochemically, neuroendocrine differentiation is not noted in the present cases.

Besides renal cell tumors, metanephric tumors have been known, which mimics developing nephron. Metanephric tumors include metanephric adenoma and metanephric adenofibroma. The former is a pure epithelial tumor composed of papillotubular architectures and a hyalinized and edematous stroma. The latter also contains a stromal element other than epithelial. Tumor cells are small in size and uniform and has high nucleocytoplasmic ratio. Immunohistochemically, the tumor cells of the metanephric tumor are positive for WT-1 [11] and CD57 [12]. Basically, metanephric tumors are benign and cured by removal of the tumor. Although the incidence is extremely rare, there is a report on metanephric adenosarcoma [13].

Metastatic thyroid papillary carcinoma is reported as a mimicry of metanephric adenoma [14]. The present case is also similar to metastatic papillary carcinoma of the thyroid. However, there was no possible primary site in the organs other than the kidney. Immunohistochemistry of TTF1 was negative. Based on these findings, the possibility of metastatic thyroid papillary carcinoma was excluded.

Finally, nephroblastoma is the most frequent pediatric renal malignancy. Histologically, it shows triphasic appearance, that is, blastemal, epithelial, and stromal elements with variable proportions. Adult cases are rare in incidence, and less than 300 cases have been reported. The diagnostic criteria of adult nephroblastoma are as follows [15]: presence of a primary renal neoplasm, presence of a primitive blastemal spindle and round cell or embryonal tubular or glomeruloid structures, absence of tumor diagnostic of RCC, and age >15 years old. The presented cases were consistent with these criteria. Immunohistochemically, the tumor shows positivities of WT-1 and CD57, similar to metanephric tumors [11, 12]. However, the present cases occur in adults as nephroblastoma, and the histology is mostly of epithelial, lacking blastemal and stromal elements. Such a kind of tumors might have been diagnosed as conventional papillary RCC. In cases of complete absence of the blastemal and stromal elements, it might be considered as a malignant counterpart of metanephric adenoma.

Together with these findings, adult nephroblastoma with overwhelming epithelial component should be recognized as a differential diagnostic candidate of papillary RCC and metanephric adenoma.
